# Muscarinic signaling influences the patterning and phenotype of cholinergic amacrine cells in the developing chick retina

**DOI:** 10.1186/1471-213X-8-13

**Published:** 2008-02-06

**Authors:** Jennifer J Stanke, Bret Lehman, Andy J Fischer

**Affiliations:** 1 Department of Neuroscience, College of Medicine, The Ohio State University, Columbus, Ohio, USA; 2 College of Optometry, The Ohio State University, Columbus, Ohio, USA

## Abstract

**Background:**

Many studies in the vertebrate retina have characterized the differentiation of amacrine cells as a homogenous class of neurons, but little is known about the genes and factors that regulate the development of distinct types of amacrine cells. Accordingly, the purpose of this study was to characterize the development of the cholinergic amacrine cells and identify factors that influence their development. Cholinergic amacrine cells in the embryonic chick retina were identified by using antibodies to choline acetyltransferase (ChAT).

**Results:**

We found that as ChAT-immunoreactive cells differentiate they expressed the homeodomain transcription factors Pax6 and Islet1, and the cell-cycle inhibitor p27^kip1^. As differentiation proceeds, type-II cholinergic cells, displaced to the ganglion cell layer, transiently expressed high levels of cellular retinoic acid binding protein (CRABP) and neurofilament, while type-I cells in the inner nuclear layer did not. Although there is a 1:1 ratio of type-I to type-II cells *in vivo*, in dissociated cell cultures the type-I cells (ChAT-positive and CRABP-negative) out-numbered the type-II cells (ChAT and CRABP-positive cells) by 2:1. The relative abundance of type-I to type-II cells was not influenced by Sonic Hedgehog (Shh), but was affected by compounds that act at muscarinic acetylcholine receptors. In addition, the abundance and mosaic patterning of type-II cholinergic amacrine cells is disrupted by interfering with muscarinic signaling.

**Conclusion:**

We conclude that: (1) during development type-I and type-II cholinergic amacrine cells are not homotypic, (2) the phenotypic differences between these subtypes of cells is controlled by the local microenvironment, and (3) appropriate levels of muscarinic signaling between the cholinergic amacrine cells are required for proper mosaic patterning.

## Background

Amacrine cells are a distinct class of retinal neuron that participate in the processing and refinement of visual information. The amacrine cells receive input from other amacrine cells and bipolar cells, release inhibitory neurotransmitters (GABA and/or glycine) at synapses that are formed with ganglion cells and other amacrine cells, and participate in retinal image processing. Amacrine cells are a highly diverse class of neuron; there may be as many as 30 distinct types [1,2]. The classical findings of Cajál [3] and additional work [4-14] suggest that there may be as many as 30 different types of amacrine cells in the avian retina. Although many studies have identified mechanisms that promote or suppress amacrine cell fate, little is known about the mechanisms involved in the differentiation of specific types of amacrine cells. The factors that instruct cells from a pool of amacrine-fated neurons to form particular types of amacrine cells remain largely unknown. In this study we use the cholinergic cells in the embryonic chick retina as a model system to study the mechanisms involved in the differentiation of one particular type of amacrine cell.

Cholinergic neurons use acetylcholine (ACh) as a transmitter and are found in the retina of all vertebrate classes. Cholinergic amacrine cells have somata located at the proximal margin of the inner nuclear layer (INL; type-I) and displaced to the ganglion cell layer (GCL; type-II) with processes confined to two strata in the inner plexiform layer (IPL) [15-23]. Type-I and type-II cholinergic amacrine cells are arrayed in a mosaic pattern with near-mirror symmetry around a horizontal plane through the IPL [18,19]. However, the patterned spacing of cells within one layer is independent of the spacing of the cells in the other layer [24]. The retinas of birds and reptiles contain a third type of cholinergic amacrine cell, with somata located near the middle of the INL and processes diffusely distributed in sub-laminae 1 through 4 of the IPL [21]. Furthermore, avian type-III cholinergic amacrine cells can be segregated into 2 subtypes: type-IIIa cells that contain enkephalin, neurotensin, and somatostatin immunoreactivities, and type-IIIb cells that do not [4].

During embryonic development, type-I and type-II cholinergic amacrine cells express ChAT (the biosynthetic enzyme that produces ACh) as early as embryonic day 6.5 (E6.5), whereas ChAT-immunoreactivity in type-III cells is not detected until 6 days prior to hatching at about E15 [23]. Cholinergic amacrine cells arise from a pool of undifferentiated post-mitotic neuronal cells and begin to differentiate in the middle of the presumptive IPL where they, coincidently, accumulate GABA [23,25]. Although a great deal is known about the morphological and temporal development of these amacrine cells, little is known about the genes that they express during differentiation and the factors that influence their development. However, it has recently been shown that extra-cellular ATP and the P2X receptor coordinate, in part, the mosaic patterning and survival of the cholinergic cells in the rodent retina [26] and that visual activity is required for maintenance of the mosaic after eye opening in the mouse [27].

The purpose of this study was to better characterize the development of type-I and type-II cholinergic amacrine cells in the chick retina and identify factors that influence their development. We find that the type-I and type-II cholinergic amacrine cells express p27^kip1^, Pax6 and Islet1 during development. In addition, we show that these cells are not homotypic during embryonic development; type-II cells transiently express elevated levels of cellular retinoic acid binding protein (CRABP) and neurofilament, while type-I cells do not. Furthermore, we show that the relative abundance of type-I and type-II cholinergic cells is influenced by local microenvironment and signalling through muscarinic acetylcholine receptors. We propose that autologous signalling between cells acts to drive the differentiation and mosaic patterning of distinct types of retinal amacrine cells.

## Results

**Figure 1:**
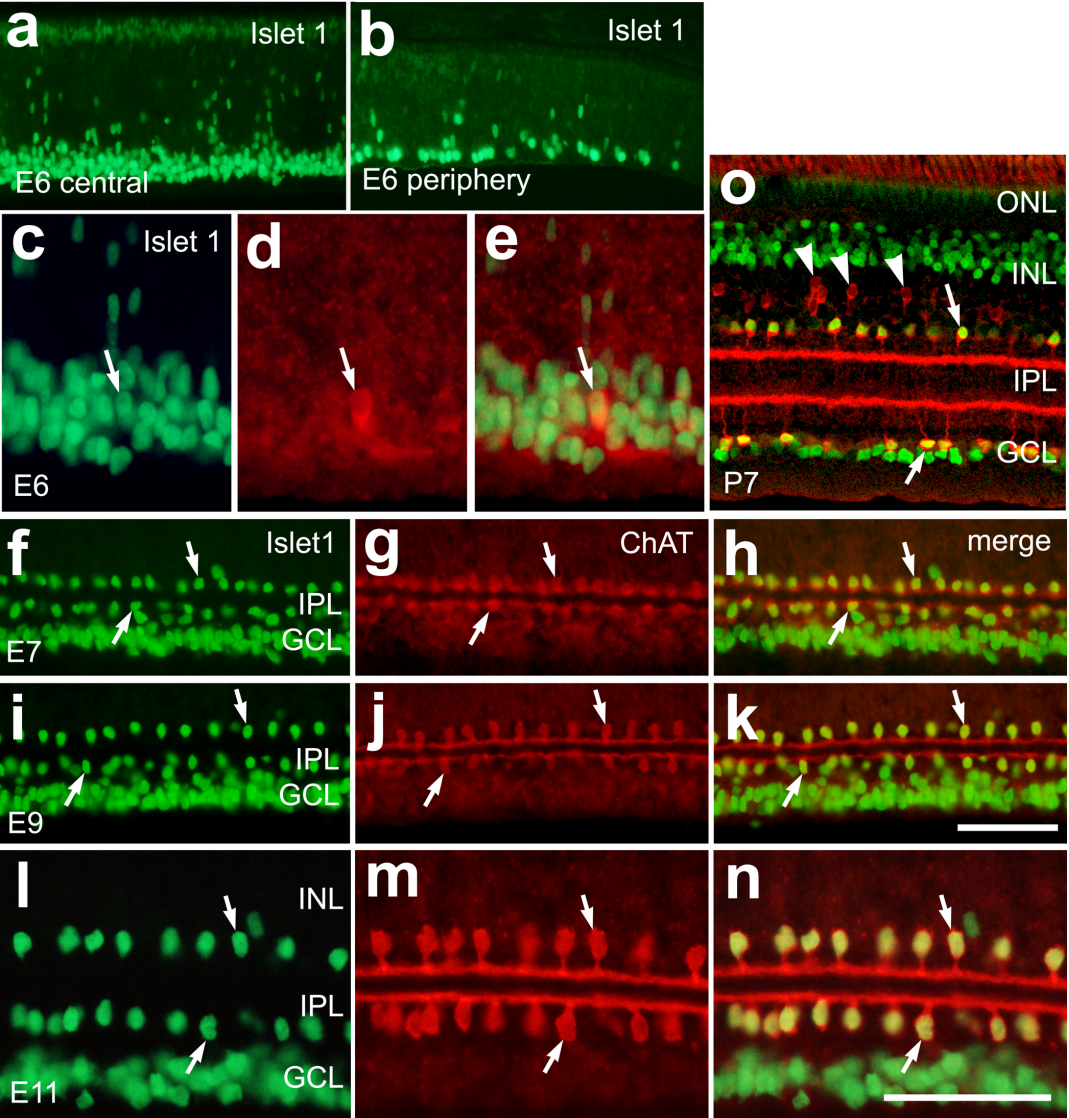
Type-I and type-II cholinergic amacrine cells express Islet1 throughout embryonic development, whereas type-III cells do not. Vertical sections of chick retina labeled with antibodies to Islet1 (green) and ChAT (red). Tissues were obtained from chicks at E6 (**a-e**), E7 (**f-h**), E9 (**i-k**), E11 (**l-n**), and P7 (**o**). Arrows indicate cells that are labeled for ChAT and Islet1, and arrow-heads indicate type-III cells that are immunoreactive for ChAT alone. The calibration bar (50 μm) in panel **k** applies to panels **a**, **b**, **f-k **and **o**, and the bar in **n **applies to panels **c-e **and **l-n**. Abbreviations: ChAT – choline acetyltransferase; INL – inner nuclear layer; IPL – inner plexiform layer; GCL – ganglion cell layer.

Cholinergic amacrine cells in the embryonic chick retina were detected by using antibodies to choline acetyltransferase (ChAT). ChAT-immunoreactive amacrine cells were first detected in the retina at embryonic day 6 (E6; Fig. 1c–e), consistent with a previous report [23]. One day later at E7, in central regions of the retina, these cells formed two uniform rows of cells, with type-I cells located at the distal border of the presumptive IPL and type-II cells located within the proximal half of the presumptive IPL (Fig. 1g), similar to previous findings [23,25].

### Cholinergic amacrine cells express Islet1 and Pax6 as they differentiate

In the developing rodent retina, cholinergic amacrine cells are known to express Islet1 [28]. Islet1 is a homeotic LIM-domain transcription factor that is known to be expressed by bipolar, horizontal, and ganglion cells in the retina [29]. In the developing avian retina at E6, Islet1-immunoreactivity was detected in the nuclei of presumptive ganglion and amacrine cells in the GCL and in differentiating cells in the distal retina (Figs. 1a and 1b). The Islet1-positive nuclei in the distal retina presumably were post-mitotic neurons migrating away from the ventricular surface of the retina to their final position within the retina. At E6, Islet1 was expressed by ChAT-immunoreactive cells prior to the formation of the presumptive IPL (Figs. 1c–e). These cells were intermingled with Islet1-expressing cells in the GCL, suggesting that the cholinergic cells begin to differentiate among a heterogeneous pool of post-mitotic Islet1+/ChAT- cells. At E7, Islet1-positive nuclei were found in the GCL, putative differentiating bipolar cells in distal layers (not shown) of the retina, and two rows of cells between the GCL and bipolar cells (Figs. 1f–h). The Islet1-positive nuclei at the vitread border of the INL and within the IPL were those of type-I and type-II cholinergic amacrine cells, respectively (Figs. 1f–h). At E9 and E11, the Islet1/ChAT-positive cells remained as distinctive rows of cells (Figs. 1i–n). By E16 the somata of the type-II cells had migrated into the GCL. In the retina of postnatal chickens, Islet1-immunoreactivity was present in horizontal cells, many bipolar cells, most if not all ganglion cells, and type-I and type-II cholinergic amacrine cells, but not type-III cells (Fig. 1o).

**Figure 2:**
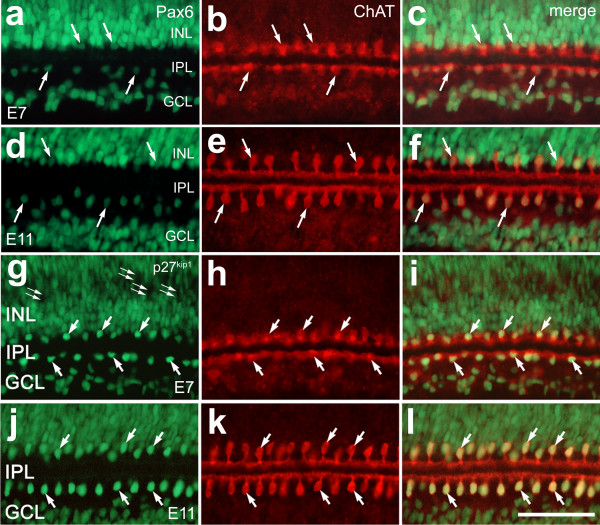
Cholinergic amacrine cells express low levels of Pax6 and high levels of p27^kip1 ^compared to other amacrine cells. Vertical sections of chick retina were labeled with antibodies to Pax6 (green; **a**, **c**, **d **and **f**) or p27^kip1 ^(green; **g**, **i**, **j **and **l**) and ChAT (red). The tissues were obtained from chicks at E7 (**a-c **and **g-i**) and E11 (**d-f **and **j-l**). Arrows indicate cholinergic amacrine cells that are labeled for Pax6 or p27^kip1^, and small double arrows indicate p27^kip1^-negative cells in the progenitor cell layer of the E7 retina. The calibration bar (50 μm) in panel **l **applies to all panels. Abbreviations: ChAT – choline acetyltransferase; INL – inner nuclear layer; IPL – inner plexiform layer; GCL – ganglion cell layer.

To test whether homeodomain transcription factors in addition to Islet1 are expressed by developing cholinergic amacrine cells, we double-labeled sections with antibodies to ChAT and Pax6, AP2α, Prox1 or Brn3b. Pax6 is paired-class homeodomain transcription factor that is known to be expressed by neural progenitors in the embryonic and postnatal chick retina [30-33]. Pax6 is expressed at low levels in progenitors and becomes expressed at high levels in amacrine and ganglion cells as they differentiate [30,31]. We used a monoclonal antibody to Pax6 that is known to label horizontal, amacrine, ganglion cells and progenitors in the avian retina [29,31,32]. In E7 and E11 retinas, type-I and type-II cholinergic amacrine cells expressed relatively low levels of Pax6; Pax6-immunolabeling was more intense in the nuclei of non-cholinergic neurons in the GCL and INL compared to the labeling intensity of the nuclei of the ChAT-immunoreactive cells (Figs. 2a–f). The cholinergic amacrine cells did not express AP2α, Prox1, or Brn3a during embryonic development or in the postnatal retina (results not shown).

The cell cycle inhibitor p27^kip1 ^has been shown to be important for the development of the retina and differentiation of Müller glia [34-36]. In E7 retina, immunoreactivity for p27^kip1 ^was seen in numerous nuclei scattered throughout the retina, but was absent from the nuclei of proliferating progenitors in distal layers of the retina (Figs. 2g–i). The distribution of p27^kip1^-immunoreactive nuclei in E7 retina was consistent with the known location of differentiating cells. At this stage of development, immature ChAT-positive cells contained p27^kip1^-immunoreactive nuclei (Figs. 2g–i). At E11, numerous p27^kip1^-positive nuclei were observed in the INL, IPL and GCL (Fig. 2j–l). The p27^kip1^-positive nuclei in the IPL were immunoreactive for ChAT, and the labeling intensity of these nuclei was greater than that seen in the nuclei of non-cholinergic cells of the INL and GCL (Figs. 2j–l).

### Cholinergic amacrine cells transiently express neurofilament and CRABP as they differentiate

**Figure 3:**
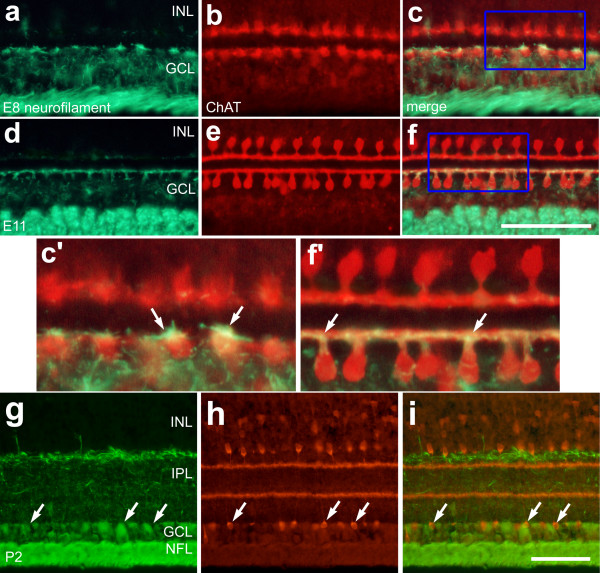
Type-II cholinergic amacrine cells transiently express neurofilament during embryonic development. Vertical sections of chick retina labeled with antibodies to neurofilament (green) and ChAT (red). Sections were obtained from embryos at E8 (**a-c**) E11 (**d-f**), or from a postnatal chick (P2) (**g-i**). The boxed-out areas in panels **c **and **f **are enlarged 2.5-fold in panels **c' **and **f'**. Arrows indicate the dendrites of type-II cholinergic amacrine cells that co-localize immunoreactivities for neurofilament and ChAT. The calibration bar (50 μm) in panel **f **applies to panels **a-f**, and the bar in **i **applies to panels **g-i**. Abbreviations: ChAT – choline acetyltransferase; INL – inner nuclear layer; IPL – inner plexiform layer; GCL – ganglion cell layer; NFL – nerve fiber layer.

Neurofilament is known to be expressed by ganglion cells soon after they begin to differentiate, and the ganglion cells in the chick retina are thought to be the only type of neuron that expresses this intermediate filament [37]. Surprisingly, we found that the neurites of type-I and type-II cholinergic amacrine cells were immunoreactive for neurofilament in the retinas from E8 (Figs. 3a–c) through E12 (Figs. 3d–f). Increased levels of neurofilament-immunoreactivity were detected in the type-II cholinergic cells, compared to levels seen in the type-I cells (Fig. 3). At E8, neurofilament-immunoreactivity was present in a "tuft" of processes at the distal edge of the type-II cholinergic amacrine cells, while little or no immunoreactivity was detected in the type-I cells (Fig. 3a–c). By E12, neurofilament-immunoreactivity was present throughout the neurites of type-II cells, while sparse immunoreactivity was present in the neurites of type-I cells (Fig. 3d–f). In E18 and postnatal retinas, neurofilament-immunoreactivity was not present in the cholinergic amacrine cells (Figs. 3g–i).

**Figure 4:**
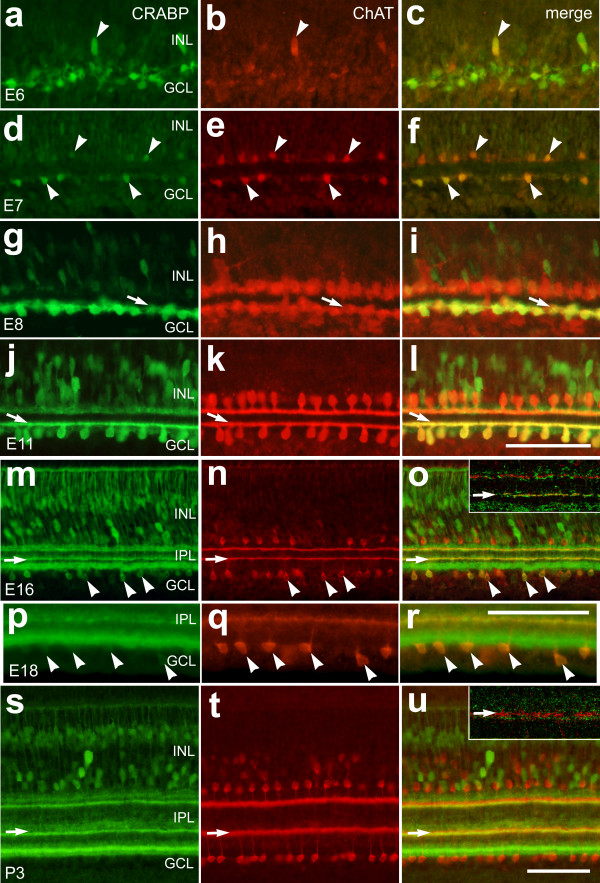
CRABP is transiently expressed by type-II cholinergic amacrine cells during embryonic development. Vertical sections of chick retina labeled with antibodies to CRABP (green) and ChAT (red). Sections were obtained from embryos at E6 (**a-c**), E7 (**d-f**), E8 (**g-i**), E11 (**j-l**), E16 (**m-o**), E18 (**p-r**) or from a postnatal chick (P3; **s-u**). Arrows indicate the IPL strata that are occupied by the dendrites of type-II cholinergic amacrine cells, and arrow-heads indicate representative cells that are immunoreactive for ChAT and CRABP. The calibration bar (50 μm) in panel **l **applies to panels **a-l**, the bar in **r** applies to **p-r**, and the bar in **u **applies to to **m-o** and **s-r**. The insets in panels **o **and **u **are high-magnification confocal images of the inner plexiform layer where the dendrites or the type-II cells terminate. Abbreviations: CRABP – cellular retinoic acid binding protein; ChAT – choline acetyltransferase; INL – inner nuclear layer; IPL – inner plexiform layer; GCL – ganglion cell layer.

In the embryonic chick retina, CRABP was expressed by numerous differentiating amacrine cells (Fig. 4), consistent with a previous report [38]. At E6, CRABP-immunoreactive cells were found near the developing GCL (Fig. 4a) and a few of these cells were also immunoreactive for ChAT (Figs. 4a–c). At E7, most of the CRABP-immunoreactive cells were located in the presumptive IPL and these cells were also immunoreactive for ChAT (presumptive type-II cholinergic cells) (Figs. 4d–f). Low levels of CRABP could be detected in a few (7.05%, n = 242) presumptive type-I cells in the proximal INL (Figs. 4d–f). This finding indicates that a minority of type-I cells may express low levels of CRABP during a narrow window of development. In E8 retina, high levels of CRABP-immunoreactivity were detected in type-II cholinergic cells located in the proximal IPL and weak CRABP-immunoreactivity was detected in non-cholinergic cells as they differentiate in the presumptive INL (Fig. 4g). The cell bodies of type-I cells contained little or no CRABP-immunoreactivity in the E8 retina. Differential expression of CRABP was most noticeable at E11, after the cells have extended their neurites into the IPL. Type-II cells expressed high levels of CRABP in their cell bodies, as well as neurites, whereas CRABP was absent from somata of type-I cells and low levels may have been present in the neurites (Figs. 4j–l). Type-II cholinergic cells continued to express CRABP as they differentiated through E16 (Figs. 4m–o). At E18, 3 days before hatching, immunoreactivity for CRABP was no longer detectable in the type-II cholinergic cells (Figs. 4p–r). To better assess whether the dendrites of the cholinergic cells contained CRABP, high-magnification confocal images were obtained. In E16 retinas, we observed significant CRABP-immunoreactivity in the stratified neurites of the type-II cells. By contrast, CRABP-immunoreactivity was no longer detectable in the processes of type-II amacrine cells at P3 (compare Fig. 4o inset and 4u inset). In postnatal chick retina, CRABP-immunoreactivity was not observed in type-II cholinergic cells (Figs. 4s–u), but was present in non-cholinergic amacrine cells and bipolar cells, consistent with a previous report [39]. Taken together, these findings indicate that during embryonic development the cholinergic cells are not homotypic. The phenotypic differences between type-I and type-II cells may occur because of differences imposed by the local microenvironment. In other words, the local microenvironment provided by the INL likely influences the phenotype of type-I cells, whereas the proximal IPL and/or GCL may influence the phenotype of the type-II cells; a spatial separation of 20 to 30 μm within the developing retina.

### Differentiation of cholinergic amacrine cells in dissociated cell cultures

**Figure 5:**
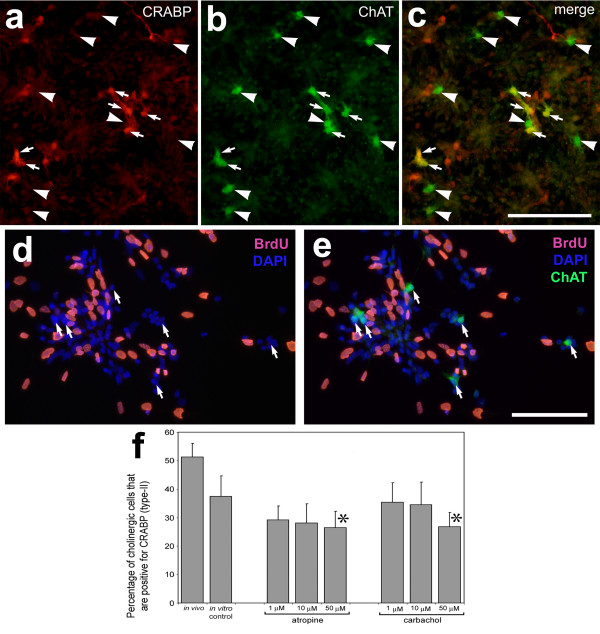
*In vitro *conditions and muscarinic signaling influence the ratio of type-I to type-II cholinergic amacrine cells. E7 retinas were dissociated, plated and grown in culture for 24 hours. After 24 hours the cells were added with atropine (1, 10 or 50 μM), carbachol (1, 10 or 50 μM), or saline as a control, and maintained in culture for an additional 48 hours. Cells were labeled with antibodies to CRABP (red) and ChAT (green). Arrows (**a-c**) indicate type-II cholinergic cells that are immunoreactive for ChAT and CRABP, and arrow-heads indicate type-I cholinergic cells that are immunoreactive for ChAT alone. Cholinergic cells (arrows, **d** and **e**) did not accumulate BrdU after 3 DIV. The calibration bar (50 μm) in panel **c **applies to **a-c **and in **e **applies to **d **and **e**. Panel **f **shows the percentage of cholinergic cells also positive for CRABP (type-II cells). Asterisks indicate p-values < 0.05. Abbreviations: CRABP – cellular retinoic acid binding protein; ChAT – choline acetyltransferase; BrdU – Bromodeoxyuridine.

To test whether the microenvironment influences the phenotype of type-I and type-II cholinergic amacrine cells, we dissociated retinas from E7 chick embryos and maintained these cells in culture for 3 days. Dissociated cell cultures will not accurately recapitulate the microenvironment provided *in vivo *where different cells, and the cues provided by these cells, are highly spatially organized into discrete laminae. To discriminate between type-I and type-II cholinergic amacrine cells we double-labeled cells with antibodies to ChAT and CRABP. Cells immunoreactive for ChAT and CRABP (in the cell body) were counted as type-II cells and cells that were immunoreactive for ChAT alone were counted as type-I cells (Figs. 5a–c). *In vivo*, numbers of type-I and type-II cholinergic amacrine cells in the developing retina are approximately equal (51.2 ± 4.8%). In dissociated cell cultures of embryonic retina, however, type-I cholinergic cells are approximately twice (2.1 ± 0.4; n = 5 culture sets) as abundant as type-II cells (Figs. 5a, 5b and 5f). The decrease in the relative abundance of type-II to type-I cells did not change significantly with differences in original plating density (50,000 to 200,000 cells/cm^2^; data not shown). Prada and colleagues [25] showed that cholinergic cells are postmitotic by about E4. To exclude the possibility of *de novo *generation of cholinergic cells in culture, we cultured E7 retina in the presence of BrdU (to label newly generated cells) for 3 days. None (n = 131) of the ChAT-positive cells in cultures of E7 retina accumulated BrdU (Figs. 5d–e), indicating that *de novo *generation of cholinergic amacrine cells does not occur under culture conditions. Taken together, these findings suggest that the environment provided by the intact retina is required to maintain equal numbers of type-I and type-II cholinergic cells.

Since the type-II cholinergic cells are in close proximity (<15 μm) to developing ganglion cells, whereas the type-I cells are further away (>30 μm), it is possible that signals provided by the differentiating ganglion cells influence the development and phenotype of the type-II cells. Ganglion cells are known to produce Sonic Hedgehog (Shh) as they differentiate [40,41] and Shh is known to establish morphogenic gradients over short distances [42,43]. Thus, it is possible that Shh stimulates type-II cholinergic amacrine cells to transiently express elevated levels of CRABP during embryonic development. However, the relative abundance of type-II cells was not significantly affected by the addition of Shh or the blockade of Shh-signaling with KAAD (3-keto, N-amino-ethyl aminocaproyl dihydrocinnamoyl) cyclopamine. Shh-treated samples contained 3.1% (± 10.8%; 151 cells) more type-II cells, and cultures treated with KAAD-cyclopamine contained 6.7% (± 5.7%; 140 cells) fewer type-II cells compared to control preparations. Statistical analyses indicated that these changes were not significant.

There is some evidence that type-II cholinergic amacrine cells express muscarinic receptors in the postnatal retina [44]. In addition, antibodies to the m2 and m4 isoforms of the muscarinic receptor in the embryonic chick retina appear to label type-II-like cells in the developing IPL [45,46]. Thus, it is possible that the type-II cholinergic amacrine cells communicate to each other through paracrine muscarinic interactions. To test this hypothesis we applied a muscarinic antagonist (atropine) or agonist (carbachol) to cultures of E7 retinas. We found that activation *and *suppression of muscarinic signaling reduced the relative abundance of type-II cholinergic amacrine cells (Fig. 5f).

Since the relative abundance of type-II to type-I cholinergic amacrine cells is reduced by *in vitro *conditions and by influencing muscarinic signaling, we sought to assess whether similar mechanisms influence the phenotype of cholinergic cells in the intact retina. To test this, we made intraocular injections of 300 ng of atropine (a muscarininc antagonist) or 200 ng carbachol (a muscarinic agonist) into the vitreous chamber of E7 chick embryos. The E7 eye was estimated to have 75 mm^3 ^of volume, which should have resulted in initial maximum vitreal concentrations of carbachol and atropine of about 25 μM. Atropine and carbachol have been used from 15 nM to 100 mM to elicit effects on retinal cells and chick muscarinic acetylcholine receptors (mAChR) [47-49].

**Figure 6:**
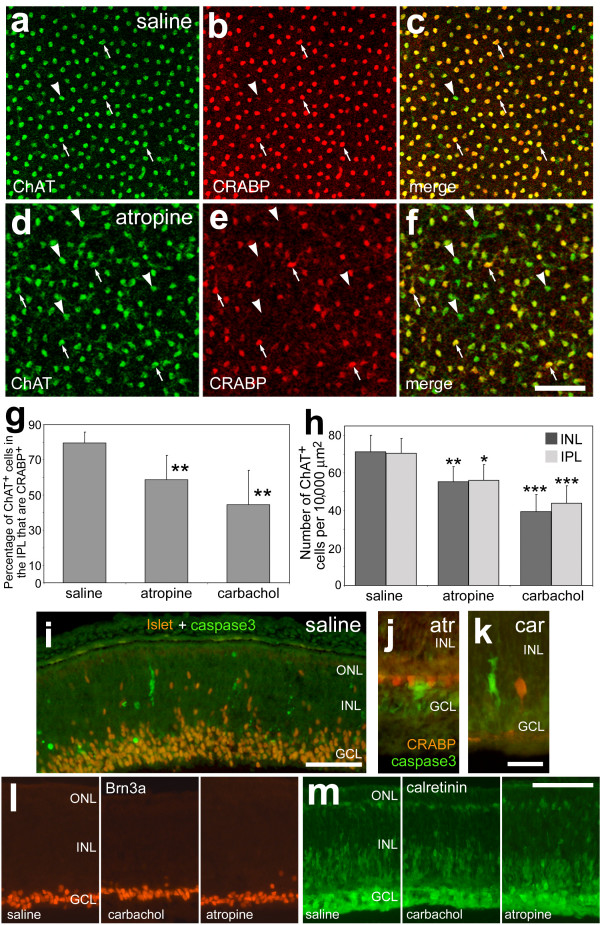
Muscarinic signaling influences the phenotype and density of cholinergic amacrine cells in the intact retina. Confocal microscopy was used to obtain optical sections of flat-mounted retinas labeled with antibodies to ChAT (green) and CRABP (red). Optical sections were obtained at the level of the developing IPL to visualize the type-II cells. Images are shown from retinas treated with saline (**a-c**) or atropine (**d-f**). The arrows indicate type-II cells that are immunoreactive for ChAT and CRABP, and the arrow-heads indicate cells that are immunoreactive for ChAT alone. Panel **g **is a histogram of the percentage of type-II cholinergic cells in the IPL that are immunoreactive for CRABP. Cells were counted from retinas that were treated with saline, atropine or carbachol. Panel **h **is a histogram of the number of ChAT-immunoreactive cells in the INL (type-I) and presumptive IPL (type-II) in retinas treated with saline, atropine or carbachol. The significance of difference was assayed by using ANOVA (p < 0.02) and a post-hoc Student's t-test (2 tailed, equal variance; *p < 0.05, **p < 0.025, ***p < 0.002). Panels **i-m **are representative microgrographs of retinal sections labeled for cleaved caspase 3 (green; **i-k**) and Islet1 (red; **i**) or CRABP (red; **j **and **k**), Brn3a (**l**), and calretinin (**m**). The calibration bar (50 μm) in panel **f **applies to panels **a-f**, the bar (50 μm) in **i **applies to **i **alone, the bar (10 μm) in **k **applies to **j **and **k**, and the bar (50 μm) in **m **applies to **l **and **m**. Abbreviations: INL – inner nuclear layer, IPL – inner plexiform layer, ChAT – choline acetyltransferase, CRABP – cellular retinoic acid binding protein.

Similar to the findings of the *in vitro *studies, we found that the number of cells that express ChAT and CRABP (type-II-like cells) was decreased by muscarinic ligands. Treatment with carbachol or atropine reduced the number of ChAT-positive cells that are immunoreactive for CRABP in the presumptive IPL (ANOVA, p = 0.017). There was a reduction in the relative abundance of ChAT/CRABP-positive cells by about 23% with atropine treatment (n = 6, p = 0.021) or by about 38% with carbachol treatment (n = 4, p = 0.018) (Fig. 6g). The abundance of type-II-like cells in the IPL was not significantly different between treatments with atropine or carbachol. We consistently found that the type-I cells in the INL did not express detectable levels of CRABP in retinas that were treated with muscarinic ligands. These findings suggest that gains *or *losses in muscarinic signaling decrease the relative abundance of CRABP-expressing cells among the type-II cholinergic amacrine cells *in vivo*.

In addition to a decrease in the percentage of type-II cholinergic amacrine cells, we found that the density of ChAT-positive cells was decreased by treatment with both a muscarinic AChR antagonist and agonist (ANOVA p = 0.0013, GCL; p = 0.0004, INL; Fig 6h). The number of ChAT+ cells per 10,000 μm^2 ^in the INL (type-I cells) and in the GCL (type-II cells) was significantly decreased by intraocular injections of atropine (p = 0.024, INL; p = 0.035, GCL; n = 6). Similarly, treatment with the muscarinic agonist carbachol decreased the density of cholinergic amacrine cells in the INL and GCL (p = 0.0011, INL; p = 0.002, GCL; n = 4). Decreases in the density of cholinergic cells were not caused by increased apoptosis; we failed to find increased numbers of cleaved caspase3-positive cells in retinas treated with atropine or carbachol (Figs. 6i–k). Additionally, the muscarinic ligands did not have any obvious effect on non-cholinergic neurons; ganglion cells labeled for Brn3a and horizontal, bipolar, amacrine and ganglion cells labeled for calretinin displayed normal distributions when treated with atropine or carbachol (Figs. 6l and 6m).

**Figure 7:**
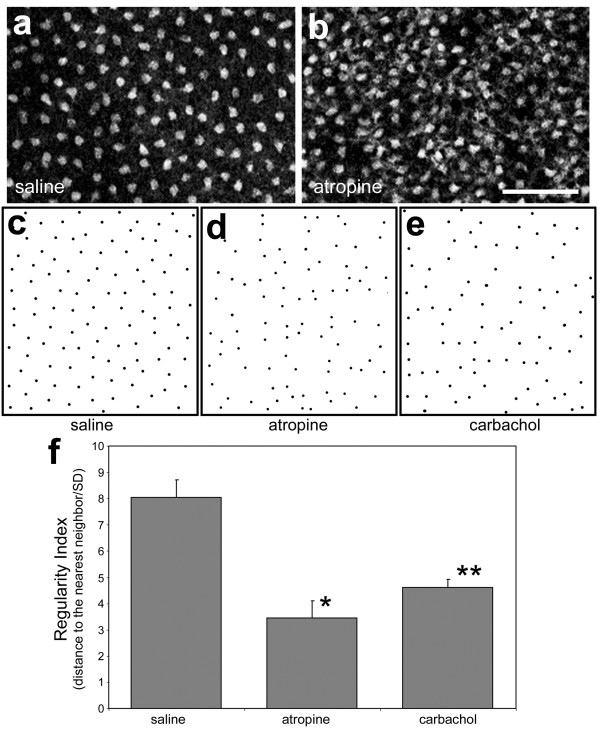
Stimulation and inhibition of muscarinic signaling disrupts the patterning of cholinergic amacrine cells. Panels **a **and **b **are representative fields of view from the GCL of retinas treated with saline (**a**) or atropine (**b**). Whole-mount preparations of the retina were labeled with antibodies to ChAT and images were obtained by using confocal microscopy. The calibration bar (50 μm) in panel **b **applies to panels **a **and **b**. Panels **c-e **are dot-plots that were obtained by marking the center of individual cholinergic cells in the GCL of retinas treated with saline (**c**), atropine (**d**) or carbachol (**e**). Panel **f **is a histrogram of the mean regularity indices measured from cells of 3 different retinas that were treated with saline, atropine or carbachol. The significance of difference was assayed by using ANOVA (p = 0.0017) and a post-hoc Student's t-test (2 tailed, equal variance; *p = 0.00016, **p < 0.0013).

In retinas treated with atropine or carbachol, we found that the regularly patterned mosaic of cholinergic amacrine cells was disrupted (Figs. 7a–e). By visual inspection, the type-I and type-II cells in retinas treated with atropine or carbachol appeared irregularly spaced compared to saline-treated retinas (Figs. 7a and 7b). To quantify whether the mosaic patterning of the cholinergic cells was disrupted by the muscarinic ligands, we performed a nearest neighbor analysis. Nearest neighbor regularity indices with values above 7 are considered to be regular and values below 4 represent a random distribution. In saline-treated retinas the regularity index of the cholinergic amacrine cells was high, with a value of 8.04 ± 0.68 (Fig. 7f). The regularity index of the cholinergic cells was significantly decreased in retinas treated with atropine (3.31, p = 0.0002) or carbachol (5.02, p = 0.0013) compared to the regularity index of the cholinergic cells in saline-treated retinas (Fig. 7f). These findings suggest that perturbation of muscarinic signaling disrupted the mosaic patterning of the cholinergic amacrine cells.

## Discussion

The genes that are expressed by differentiating cholinergic amacrine cells are distinctly different from those that are expressed by other types of amacrine cells. Unlike other types of amacrine cells, the cholinergic cells express Islet1 with the onset of differentiation and into the mature retina, consistent with reports in the embryonic rat retina [28]. As the cholinergic amacrine cells differentiate they express proteins common to other types of amacrine cells such as Pax6, CRABP and p27^kip1^. However, the relative levels of immunolabeling for these proteins within the cholinergic cells are either decreased (Pax6) or increased (CRABP and p27^kip1^) compared to levels seen in other types of differentiating amacrine cells. It is likely that Islet1 and Pax6 play important roles in the development and maintenance of phenotype of the type-I and type-II cholinergic amacrine cells. The significance of elevated expression of p27^kip1 ^and CRABP in the cholinergic cells remains unknown.

The type-I and type-II cholinergic amacrine cells are equally abundant, morphologically identical, and are arranged with near-mirror symmetry across a horizontal plane through the IPL [18,19]. In addition to using acetylcholine, type-I and type-II cholinergic amacrine cells are known to utilize GABA as a neurotransmitter [50,51] and begin to accumulate GABA soon after they begin to differentiate [25]. Furthermore, type-I and type-II cholinergic amacrine cells in the chick retina have been shown to originate from a common pool of migrating, post-mitotic undifferentiated neurons [25]. These findings have lead to the assumption that these cells are phenotypically identical and differ only by the position of their somata. However, we provide evidence that these two cell types differ by more than the location of their cell bodies. We found that during differentiation, type-II cells transiently expressed high levels of CRABP and neurofilament, whereas the type-I cells do not. Further, in the postnatal chick retina, type-II cells may express mAChR4, whereas type-I cells do not [44,52], and these cells have different sensitivities to excitotoxins, suggesting that different types of glutamate receptors are expressed by these cells, and/or that they have different susceptibilities to large fluctuations in ion concentrations [4]. Embryonic expression of mAChR2, mAChR3, and mAChR4 appear in non-overlapping laminae of the developing IPL at E9 [52], suggesting that these receptors may be expressed by different types of amacrine cells. The mAChR1 may not exist in the chick and [53] the retinal expression of mAChR5 [54] remains uncertain, but has been detected in the sclera of Guinea pigs [55,56] and human [57]. Differential expression of receptor isoforms is a possible mechanism to explain the differing effects of muscarinic ligands on the type-I and type-II cholinergic cells.

The cholinergic cells may be among the first types of amacrine cell to differentiate, with the onset of ChAT and Islet1 expression occurring while these cells are interspersed among the developing ganglion cells at E6. By comparison, amacrine cells that utilize different neurotransmitters such as dopamine, glucagon, enkephalin or vasoactive intestinal peptide differentiate 6–9 days after the cholinergic cells [58]. As development proceeds, the Islet1/ChAT-positive cells migrate away from the ganglion cells into the presumptive IPL where they begin to differentiate. When the type-I and type-II cholinergic cells become segregated into different laminae, the type-II cells begin to express elevated levels of CRABP and neurofilament. The close proximity of type-II cells to developing ganglion cells may expose these cells to environmental cues that influence the differentiation of ganglion cells. Accordingly, this microenvironment may promote the expression of genes common to both ganglion and type-II cells (including Pax6, Islet1 and neurofilament). Secreted signals produced by ganglion cells that influence the type-II cholinergic cells could include Shh [40,41] and BMP2/7 [59]. However, we failed to find evidence that Shh influences the differentiation of type-II cholinergic cells.

We found that the 1:1 ratio of type-I to type-II cholinergic cells from the intact retina was disrupted when the cells were dissociated and grown in culture; type-I cells were twice as abundant as the type-II cells *in vitro*. In our *in vitro *paradigm, it is likely that CRABP expression is down-regulated by the type-II cells making them indistinguishable from the type-I cells. The disruption of the *in vivo *microenvironment did not favor the differentiation of type-II cholinergic cells – the co-expression of ChAT and CRABP. These findings suggest that the factors promoting the type-II phenotype are diluted under culture conditions and that the phenotype of type-II cells relies, in part, on the microenvironment provided by the intact retina. Alternatively, the survival of type-II cholinergic amacrine cells may be selectively compromised by acute dissociation and culture conditions (i.e. serum). It is unlikely that changes in the relative abundance of type-I to type-II cholinergic cells resulted from cell fate decisions made during the terminal mitosis. We did not find any cholinergic cells in our *in vitro *and *in vivo *paradigms that were labeled for BrdU, indicating that none of the cholinergic cells were generated after the cultures were established or after compounds were delivered to the developing eye.

Similar to the results of our *in vitro *studies, we found that the *in vivo *development of the cholinergic amacrine cells was influenced by muscarinic ligands. In saline-treated retinas, the vast majority of cholinergic cells in the GCL express CRABP. However, treatment with muscarinic ligands resulted in increased numbers of type-II cells that failed to express CRABP. These findings suggest that appropriate levels of muscarinic signaling are required for the maintenance of CRABP expression in the type-II cells; perturbation of optimal levels of muscarinic signaling between cholinergic cells by agonist or antagonist disrupted the phenotype of these cells. It remains uncertain whether the effects of the muscarinic ligands were mediated by direct or indirect actions on the type-II cells. The patterning of the cholinergic amacrine cells is likely coordinated by several distinct signaling pathways in addition to the muscarinic pathway. For example, extra-cellular ATP and the P2X receptor influence the patterning and survival of the cholinergic cells in the rodent retina [26]. In addition, Zhang and colleagues [60] have shown that maintenance of array of type-II amacrine cells in the mouse GCL is dependent upon visual activity; after 30 days of rearing pups in the dark, ChAT-positive cells are undetectable in the GCL, but remain present in the INL.

Inhibiting mAChRs with atropine or activating mAChRs with carbachol disrupted the regular spacing between the cholinergic amacrine cells. We propose that the proper patterning of the cholinergic cells requires optimal levels of muscarinic signaling similar to the Shh concentration-dependent differentiation of spinal cord interneurons [61]. In principle, release and diffusion of ACh from a cholinergic cell should result in a concentration gradient that decreases with increasing distance from the source. Regular spacing between cells can occur if like-cells are repelled from each other; and for the cholinergic amacrine cells this may involve migrating laterally away from neighboring, concentrated sources of ACh. Our data are consistent with the hypothesis that the regular spacing of the cholinergic cells requires an optimal level of muscarinic signaling, and if these cells encounter gains or losses in signaling the mosaic patterning is disrupted.

It remains uncertain what effect perturbations in muscarinic signaling have on the spontaneous calcium waves, spread through the cholinergic amacrine cells, that are necessary for the establishment of early retinal circuitry in the IPL [27,62]. The mosaic pattern of the cholinergic cells may, in part, be required to establish normal waves of activity. However, this activity was shown to be regulated by nicotinic AChR in the embryonic retina [27,63]. It is possible that the application of muscarinic compounds generally perturbed retinal development and thereby disrupted the patterning of the cholinergic cells. However, we found that muscarinic compounds did not affect non-cholinergic cells expressing calretinin, Islet1 or Brn3a-positive ganglion cells, and we failed to observe an increase in immunolabeling for cleaved-caspase 3. Taken together, these findings indicate muscarinic ligands do not overtly affect non-cholinergic inner retinal neurons.

The type-I and type-II cells are not likely to influence each other through cholinergic signaling across the developing IPL. For example, the mosaic pattern of type-II cholinergic amacrine cells in the GCL has no relation to the pattern of type-I cells in the INL [24]. Additionally, acetylcholine esterase (AChE) is first detected in the chick retina between E7 and E9 in the middle of the IPL when the differentiating cholinergic cells are segregating between the GCL and INL [48,64]. AChE that is distributed into discrete laminae in the IPL should prevent any vertical diffusion of ACh through the retina. For example, ACh released from the type-II cells is not likely to act on type-I cells because AChE in the IPL is stratified between the dendrites of these cells and should degrade the ACh that diffuses distally through the IPL. However, given the dense intermingling of the dendrites of the cholinergic cells within narrow strata of the IPL, it seems likely that ACh released from the dendrites of one cholinergic cell could act on a neighboring cell with overlapping, co-stratified dendritic arbors.

It is possible that decreases in the density of cholinergic cells resulted from increases in cell death. However, we failed to detect increased numbers of cleaved-caspase 3 positive cells in retinas treated with carbachol or atropine. We cannot exclude the possibility that the death of cholinergic cells occurred in a caspase 3-independent manner resulting in reduced numbers of cholinergic cells and perhaps disruption of the mosaic patterning. Another possibility is that decreases in the abundance of ChAT/CRABP-positive cells in the IPL resulted from the down-regulation of ChAT and CRABP. Consistent with this hypothesis, ChAT may be transiently expressed by some cells in the GCL similar to the developing turtle retina, indicating that ChAT expression may be reversible in some types of retinal cells [65].

The large diversity of amacrine cell types that are distributed in mosaic patterns with regular spacing implies that autologous signals are required to coordinate the patterning of unique cell types. The notion of homotypic interactions underlying mosaic patterning has been proposed for the cholinergic amacrine cells in the retina [65]. Furthermore, Rossi and colleagues [66] demonstrated that the establishment of regular spacing between horizontal cells in the rodent retina occurs independent of pre-synaptic input from photoreceptors, suggesting that patterning is mediated via homotypic interactions among the horizontal cells. It is possible that homotypic interactions are responsible for the regular spacing of the different types of horizontal, amacrine and ganglion cells. Consistent with this hypothesis, cones, horizontal cells, ganglion cells, and cholinergic amacrine cells migrate laterally away from their radial column of clonally derived cells, whereas bipolar cells and Müller glia do not [67]. To establish a highly ordered mosaic pattern, cells within the retina must have the ability to migrate laterally away from columns of clonally derived cells unless the order of the array is determined by the progenitors. A likely mechanism underlying the spatial patterning between like-cells is the use of peptide or amino acid-derived transmitters to provide homotypic signals between cells. For example, dopamine, glucagon, neuropeptide Y, substance P, vasoactive intestinal polypeptide or enkephalin, neurotransmitters that are used by distinct types of amacrine cells, are used as autologous signals to coordinate cell type-specific patterning. Consistent with this hypothesis, our data indicate that the disruption of muscarinic ACh signaling perturbs the numbers, spacing and phenotype of cholinergic amacrine cells in the developing retina.

## Conclusion

We conclude that, during development, type-II cholinergic amacrine cells transiently express CRABP and neurofilament, while type-I cells do not. We propose that the transient expression of CRABP and neurofilament is regulated by local microenvironment. Additionally, we find that interfering with muscarinic signaling decreases the relative number of type-II cells and mosaic patterning of the cholinergic amacrine cells. We propose that the phenotypic differences between developing type-I and type-II cholinergic amacrine cells are elicited, in part, by paracrine cholinergic signaling; the local microenvironment of the in tact retina allows precise muscarinic signaling to establish proper cellular phenotypes and organization of the type-II cells. It remains uncertain whether muscarinic receptors are differentially expressed by the type-I and type-II cells.

## Methods

### Animals

The use of animals in these experiments was in accordance with the guidelines established by the National Institutes of Health and the Ohio State University. Eggs were obtained from the Department of Animal Sciences at the Ohio State University. Chick embryos were staged according to guidelines established by Hamburger and Hamilton [68]. Newly hatched leghorn chickens (*Gallus gallus domesticus*) were obtained from the Department of Animal Sciences at the Ohio State University and kept on a cycle of 12 hours light, 12 hours dark (lights on at 7:00 am). Chicks were housed in a stainless steel brooder at about 27°C and received water and Purina™ chick starter *ad libitum*.

### Cell Culture

Embryonic chick retinas were dissected in sterile Hanks' buffered saline solution added with 3% D-glucose and 0.01 M HEPES buffer (HBSS+). Retinal cells were dissociated by mild trituration after a 10-minute incubation at 37°C in Ca^2+^/Mg^2+^-free HBSS plus 0.025% trypsin. Cell density was determined by using a hemocytometer. Between 50,000 and 200,000 cells were added to wells of a 24-well plate coated with poly-D-lysine (Sigma). Cell cultures were maintained at 37°C in 5% CO_2 _under culture medium (DMEM:F12 without glutamate or aspartate, plus 100 units/ml penicillin, 100 mg/ml streptomycin, 0.05 M HEPES; Sigma, and 1% fetal bovine serum; Invitrogen). Cultures were maintained for 1 to 4 days with 50% of the medium replaced every 24 hours. After one day *in vitro*, retinal cells were added with 20 ng/ml Sonic Hedgehog (rmShh-N; R & D Systems), or 40 ng/ml KAAD (3-Keto-N-amino ethyl amino caproyldihydrocinnamoyl Cyclopamine; Toronto Research Chemicals), 1, 10 or 50 μM of atropine (Sigma) or 1, 10 or 50 μM of carbachol (Sigma). Additional cultures were maintained for 3 DIV in the presence of 1 μg/ml Bromodeoxyuridine (BrdU). Cells were fixed and processed for immunocytochemistry at 1 or 3 days after the addition of Shh, cyclopamine, atropine, or carbachol.

### In ovo injections

Time-staged eggs at E6 were windowed and access to the eye obtained by carefully tearing the chorioallantois and amniotic membranes around the embryo. The stage of the embryo was verified at this time according to the guidelines established by Hamburger and Hamilton (1951). A 40 gauge wire was used to pierce the dorsal side of the right eye. A pulled capillary pipette was then used to deliver 2 μl of a solution that contained 1 mM (200 ng) carbachol, 1 mM atropine (300 ng), or sterile saline solution (control) added with 0.4% fast green in glycerol water (1:1) and 3 μg BrdU. The chorionic membranes were placed back over the embryo, the window sealed with transparent tape, and the egg placed back into the incubator. Embryos were sacrificed 24 hours later and the injected eye enucleated. The anterior portion of the eye and vitreous were removed and discarded prior to fixation. After fixation, the ventral portion of the retina was removed and labeled for BrdU to verify the efficacy of the injection and to verify that the injection was made after terminal mitosis of the cells fated to differentiate as cholinergic amacrine cells. Retinas that labeled for BrdU in the GCL or presumptive IPL were discarded from the experiments. The remaining retina was processed for whole-mount immunolabeling.

### Fixation and sectioning

Eyes were enucleated, the anterior portion of the eye dissected away, and the vitreous removed. Eyes or dissociated cells were fixed for 30 minutes at 20°C in 4% paraformaldehyde plus 3% sucrose in 0.1 M phosphate buffer, pH 7.4. Fixation was followed by 3 washes in PBS (phosphate-buffered saline; 0.05 M phosphate buffer, 195 mM NaCl, pH 7.4). Eye-cups were cryoprotected in PBS plus 30% sucrose, soaked in embedding medium (O.C.T.-compound; Tissue-Tek) for 30 minutes, and freeze-mounted onto aluminum sectioning blocks. Transverse sections nominally 12 μm thick were cut consistently from the posterior pole of the eye, near the dorsal portion of the ventral fissure (or pecten in postnatal eyes), and thaw-mounted onto SuperFrost Plus™ slides (Fisher Scientific). Sections were air-dried and stored at -20°C.

### Immunocytochemistry

Sections were washed 3 times in PBS, covered with primary antibody solution (Table 1; 150 μl of antiserum diluted in PBS plus 5% normal goat serum, and 0.2% Triton X-100), and incubated for about 24 hours at 20°C in a humidified chamber. The slides were washed 3 times in PBS, covered with secondary antibody solution (150 μl of 1:1000 Alexafluor-conjugated secondary antibodies, Invitrogen), and incubated for about 1 hour at 20°C in a humidified chamber. Finally, samples were washed 3 times in PBS and coverslip mounted with 4:1 (v/v) glycerol to water for observation under an epifluorescence microscope. When double labeling with BrdU, the complimentary label was fixed after the secondary antibody then acid treat before addition of the primary antibody to BrdU as described previously [69]. To permeablize retinas for whole-mount labeling procedures, samples were frozen (-80°C) and thawed (20°C) three times prior to incubation with the antibody solution. Both primary and secondary antibodies were incubated over night. We evaluated antibody specificity mainly by comparison with the results of previous studies using these antibodies and, where possible, by known homologies between the immunizing proteins and the chick counterparts.

**Table 1:** Antigen, species, immunogen, working dilution, and source of the antibodies used in the current study.

**Antigen**	**Species**	**Clone/catalogue#/Supplier**	**Working dilution**
AP2α	mouse	3B5/Developmental Studies Hybridoma Bank	1:50
Bromdeoxyuridine (BrdU)	rat	BUI/75/Accurate Chemicals and Scientific Corporation	1:400
Brn3a	mouse	MAB1585/Chemicon	1:1500
Choline acetyltransferase (ChAT)	rabbit	1456/Dr. Miles Epstein, University of Wisconsin	1:1500
Calretinin	Rabbit	7699/4/SWANT	1:1000
Cellular Retinoic Acid Binding Protein (CRABP) (CRABP)	mouse	C1/Dr. Jack Saari, University of Washington	1:1000
Islet1	mouse	40.2D6/Developmental Studies Hybridoma Bank	1:50
Neurofilament (NF) (160 kDa)	mouse	RMO270/Dr. V. Lee, University of Pennsylvania	1:2000
p27^kip1^	mouse	Clone 57/Transduction Laboratories	1:200
Pax6	mouse	PAX6/Developmental Studies Hybridoma Bank	1:50
Prox1	rabbit	Dr. Stanislov Tomarev, NIH	1:800

### Photography, measurements, cell counts, and statistical analyses

Photomicrographs were taken by using a Leica DM5000B microscope equipped with epifluorescence and a Leica DC500 digital camera. Confocal microscopy was done by using a Zeiss LSM 510 meta at the Campus Microscopy and Imaging Facility at Ohio State University. Confocal stacks of images were obtained for 1 μm-thick optical sections by using a 20× objective (0.75 NA) and multi-track, narrow-pass emission filter settings to exclude the possibility of fluorescence bleeding across channels. Images were optimized for color, brightness and contrast, and double-labeled images overlaid by using Adobe Photoshop™6.0. To avoid the possibility of region-specific differences within the retina, cell counts were consistently made from the same region of retina for each data set. Significance of difference among data sets was determined by using ANOVA. Data from treated and control eyes were compared statistically with the appropriate post-hoc Student's t-test.

Cell counts from cultured cells were performed on at least 100 cells from each coverslip per condition (n ≥ 5; untreated, Shh, KAAD, atropine and carbachol). Experiments were repeated three times. For whole mount images, one optical slice from a confocal z-series was selected for the INL and one for the GCL per individual. These slices were overlaid in Adobe Photoshop 6.0 and five 200 μm^2 ^fields per retina were counted. At least four individual retinae and at least 600 cells per retina were counted per condition. As described elsewhere [70,71], an index of regularity was calculated as the mean distance to the nearest neighbor divided by the standard deviation. The distance between cells was measured from confocal micrographs and was taken as the distance from the center of one cell to the center of the next nearest cell. Measurements were made from ten randomly selected cells per field of view for three individual retinas per condition (90 cells total). Data is presented as the mean and standard deviation.

## Authors' contributions

JJS performed experiments and participated in project design, data acquisition and writing the manuscript. BL assisted with *in ovo *injections as well as data acquisition. AJF conceived the project, performed experiments, acquired data and contributed to writing the manuscript. All authors have approved the manuscript.
